# A duplicated *amh* is the master sex-determining gene for *Sebastes* rockfish in the Northwest Pacific

**DOI:** 10.1098/rsob.210063

**Published:** 2021-07-14

**Authors:** Weihao Song, Yuheng Xie, Minmin Sun, Xuemei Li, Cristín K. Fitzpatrick, Felix Vaux, Kathleen G. O'Malley, Quanqi Zhang, Jie Qi, Yan He

**Affiliations:** ^1^ MOE Key Laboratory of Molecular Genetics and Breeding, College of Marine Life Sciences, Ocean University of China, Qingdao 266003, People's Republic of China; ^2^ Department of Chemistry and Biochemistry, University of California San Diego, La Jolla, CA 92093, USA; ^3^ State Fisheries Genomics Lab, Coastal Oregon Marine Experiment Station, Department of Fisheries and Wildlife, Hatfield Marine Science Center, Oregon State University, Newport, OR, USA; ^4^ Department of Zoology, University of Otago, Dunedin, New Zealand

**Keywords:** sex determination, *amh*, sex marker, gene duplication, *Sebastes* rockfishes, resequencing

## Abstract

Teleost fish are the most diverse group of vertebrates and provide opportunities to study the evolution of sex determination (SD) systems. Using genomic and functional analyses, we identified a male-specific duplication of *anti-Müllerian hormone* (*amh*) gene as the male master sex-determining (MSD) gene in *Sebastes schlegelii*. By resequencing 10 males and 10 females, we characterized a 5 kb-long fragment in HiC_Scaffold_12 as a male-specific region, which contained an *amh* gene (named *amhy*). We then demonstrated that *amhy* is a duplication of autosomal *amh* that was later translocated to the ancestral Y chromosome. *amha* and *amhy* shared high-nucleotide identity with the most significant difference being two insertions in intron 4 of *amhy*. Furthermore, *amhy* overexpression triggered female-to-male sex reversal in *S. schlegelii*, displaying its fundamental role in driving testis differentiation. We developed a PCR assay which successfully identified sexes in two species of northwest Pacific rockfish related to *S. schlegelii*. However, the PCR assay failed to distinguish the sexes in a separate clade of northeast Pacific rockfish. Our study provides new examples of *amh* as the MSD in fish and sheds light on the convergent evolution of *amh* duplication as the driving force of sex determination in different fish taxa.

## Graphical abstract

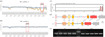

## 1. Introduction

Teleost fish are the largest and most diverse group of vertebrates and provide many opportunities to study the evolution of sex determination (SD) systems. SD mechanisms of teleost fish can be divided into three types: genetic SD (GSD), environmental SD (ESD), and a combination of GSD and ESD [1]. In GSD systems, master sex-determining (MSD) genes are thought to play a crucial role in gonad differentiation by regulating the expression of other genes. After much effort in recent decades, a few MSD genes have been identified in fish, such as *dmrt1* in the medaka species *Oryzias latipes* [2], *O. curvinotus* [3], Chinese tongue sole (*Cynoglossus semilaevis*) [4,5], *sdY* in rainbow trout (*Oncorhynchus mykiss*) [6], *gsdf* in the medaka species *O. luzonensis* [7] and breast cancer anti-resistance 1 (*BCAR1*) gene in channel catfish (*Ictalurus punctatus*) [8]. A male-specific duplication of *anti-Müllerian hormone* (*amh*) has also been identified as an MSD gene in Patagonian pejerrey (*Odontesthes hatcheri*) [9], Nile tiplapia (*Oreochromis niloticus*) [10] and northern pike (*Esox lucius*) [11]. Beyond identification of specific sex-determining genes, single-nucleotide polymorphisms (SNPs) within genes have also been reported to be responsible for SD in some fish, such as *amhr2* in fugu (*Takifugu rubripes*) [12] and *Hsd17b1* in *Seriola dorsalis* [13].

In contrast with mammals and birds, in which almost all species share the same SD systems (XX/XY in mammals and ZZ/ZW in birds), teleost fish have evolved many different SD systems. These SD systems can vary even among closely related species, as found in genus *Oryzias* [14–17], and sometimes even among different populations or lineages within a species, as in the southern platyfish (*Xiphophorus maculatus*) [18] and Nile tilapia [19]. As SD systems and MSD are not well conserved among teleosts, it is a challenge to infer evolutionary patterns and conserved themes from one species to another. However, a recent study investigated the evolution of SD in Esociformes and discovered that the northern pike MSD gene evolved from a gene duplication that occurred before 65 Mya, which has remained sex-linked on undifferentiated sex chromosome for at least 56 Mya (although a few species and populations have undergone an SD transition) [11]. In addition, a duplicated Y-specific *amhy* was associated with the male phenotype in *Odontesthes* silversides [20]*.* These results suggest that SD systems are conserved in some clades of teleost fishes.

The rockfish genus *Sebastes* is highly diverse and includes approximately 110 species worldwide [21], most of which inhabit the north Pacific Ocean, concentrated predominantly around an Asian centre near Japan and a North American centre off the coast of California [22]. *Sebastes* species exhibit great diversity in body colour, ecology, behaviour and maximum lifespan, which has made them the focus of substantial evolutionary and conservation research [21,23]. The evolution of viviparity in this genus has also long fascinated scientific curiosity [24]. In some species, older and larger females exhibit higher fecundity and therefore fisheries management requires sex identification for increased efficacy [25]. Despite the significant phenotypic variation among rockfish taxa, it is often difficult to phenotypically identify sex, and consequently researchers and fisheries managers must either distinguish the shape of male and female urogenital papillae in sexually mature adults [23,26,27], or conduct lethal dissection and examine of gonads. Therefore, identification of a genetic sex marker would be extremely useful for the improved management and conservation of rockfishes, and it would allow researchers to monitor environmental effects on SD.

SD in *Sebastes* remains poorly understood. Previous research indicates that temperature affects sex differentiation in *Sebastes*, but results have been contradictory. A study by Lee *et al*. [28] found that high temperatures resulted in a male-dominant population of *S. schlegelii*, whereas a later study of the same species found the opposite result [29]. Moreover, an entirely female population was induced by high temperature in oblong rockfish *S. oblongus* [30]. Research on GSD mechanisms in *Sebastes* has yielded similarly mixed results. A previous study identified 33 candidate male-specific markers in two rockfishes, *S. chrysomelas* and *S. carnatus,* using double digest restriction site-associated DNA sequencing (ddRAD-seq), and a PCR restriction fragment length polymorphism (PCR-RFLP) assay developed from one of these markers was able to identify sex in both species [31]. However, this PCR-RFLP assay did not successfully identify sex in six other *Sebastes* species, but rather was species-specific [32]. So far, no MSD gene has been identified in *Sebastes* species due to the lack of well-developed reference genomes [31,33].

The black rockfish (*Sebastes schlegelii*) inhabits the coasts of Japan, South Korea and China [34,35] and supports an important commercial fishery [36]. As a viviparous species, sexes can be easily identified by the appearance of external genitalia in sexually mature males. In addition, *S. schlegelii* exhibits sexual dimorphic growth, with females growing about 25% faster than males. A cytogenetic study has revealed a diploid number of 48 chromosomes, but no morphologically distinguishable sex chromosome [37]. Observations on the sexually dimorphic expression patterns of two candidate SD genes *dmrt1* and *sox3* provided no evidence for their roles in SD [38,39], and the MSD of *S. schlegelii* remains elusive. The availability of a chromosome-level genome of *S. schlegelii* [40] provides an ideal opportunity to search for an MSD.

In this study, we used resequencing and functional analysis to identify a duplicated *amh* from a male-specific region, which functions to drive testis differentiation, as a candidate male MSD gene for *S. schlegelii*. We further investigated the conservation of this putative MSD gene by PCR amplifying and Sanger sequencing the same region in three *Sebastes* species from the northwest Pacific Ocean. We also PCR amplified the same region in seven species of rockfish from the northeast Pacific Ocean, which represent a different evolutionary clade within *Sebastes* [21].

## 2. Results

### 2.1. Identification of two copies of *amh* in *Sebastes schlegelii*

**Figure 1.**
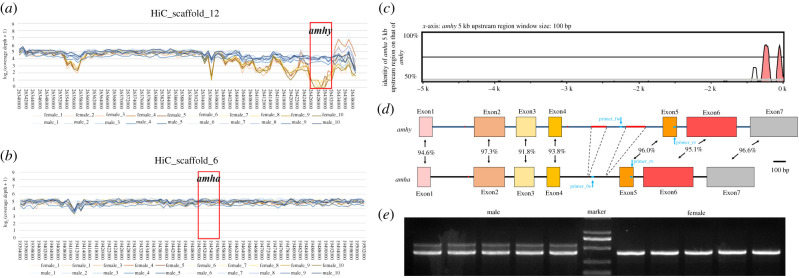
Identification of two copies of *amh* genes and exploitation of a sex marker in *S. schlegelii*. (*a*) Visualized log_2_ coverage depth for resequencing data of HiC_scaffold_12 (partial). The log_2_ coverage of the region that contained *amhy* was obviously low in female. (*b*) Visualized log_2_ coverage depth in the region of HiC_scaffold_6 (partial), which contained *amha* showed equal depth both in female and male. (*c*) Sequence alignment between 5 kb upstream region of *amha* and *amhy*. The start codon ATG was positioned at 0. (*d*) Schematic gene structure of *amha* and *amhy* from the start to stop codon. A pair of primers designed to distinguish genetical sexes is labelled. (*e*) PCR amplification produced two bands in male but only one in female.

A total of 508.66 G clean data was retained for all the samples, ranging from 17.69 G to 31.04 G for each sample, more than 98% of which were mapped to the *S. schlegelii* genome (electronic supplementary material, table S2). A DNA segment about 5 kb long on HiC_scaffold_12 was identified as a male-specific region where no reads could be detected from the females covering this area (figure 1*a*). An *amh* gene was identified in this region, which was named as *amhy* (Y chromosome-specific *amh*). Using whole-genome blast search [41], another *amh* gene was identified on HiC_scaffold_6. This *amh* gene showed high similarity with the *amhy* gene, with shared nucleotide identity ranging from 91.8% to 97.3% between exon sequences (figure 1*d*). The most significant differences between the two genes were two insertions of 131 bp and 166 bp in intron 4 (figure 1*d*) of *amhy*. The predicted proteins for *amha* and *amhy* both comprised 530 amino acids, which included the typical C-terminal TGF-β domain (amino acids 438–530) with seven canonical cysteine residues (electronic supplementary material, figure S1). Amino acid identity of the two proteins was 92.1% for the entire protein, 91.3% for the AMH_N domain and 94.6% for the TGF-β domain. In addition, the coverage depth of the region containing the *amh* gene on HiC_scaffold_6 displayed no differences in male and female (figure 1*b*). Thus, *amh* on HiC_scaffold_6 was named as autosomal *anti-Müllerian hormone* (*amha*).

### 2.2. Sex-marker exploitation

The specific insertions in intron 4 of *amhy* provided an opportunity to develop a sex marker. A pair of primers spanning the insertion of 166 bp were designed and optimized for PCR amplification using genomic DNA. The PCR assay was tested on *S. schlegelii* and it successfully distinguished males with two bands and females with one band (figure 1*e*). Sanger sequencing showed that the longer PCR product in males was from *amhy*, whereas the shorter band in males and the single PCR product in females were from *amha* (electronic supplementary material, figure S2). These results indicate that *amhy* is indeed male-specific in *S. schlegelii*.

### 2.3. Expression analysis of *amha* and *amhy*

**Figure 2.**
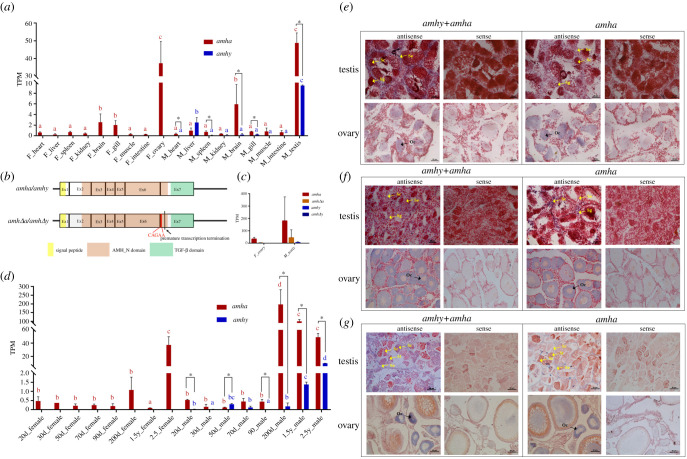
Expression pattern of *amh* genes in tissues of *S. schlegelii*. (*a*) *amhy* was predominantly expressed in testes and expressed at a low level in male liver and brain. No transcripts of *amhy* were detected in any of the female tissues. *amha* displayed significantly higher expression in the gonads compared to other tissues. Different letters mark the significant differences (*p* < 0.05) of *amha* or *amhy* expression among different tissues. Asterisk indicates the significant differences (*p* < 0.05) of expression between *amha* and *amhy* in the same tissue. (*b*) Schematic structure of alternative transcripts of *amha* and *amhy*. There was a 5 bp insertion in exon 7 causing a premature transcription termination. (*c*) Both *amhy* and *amha* displayed significantly higher expression in testes compared to ovaries. The expression of the transcripts with TGF-β domain was always higher than that of the transcripts without. (*d*) *amhy* was expressed in male samples starting from 20 dpp and peaked at 50 dpp during the sex-determining period of development. Expression of *amhy* increased with the maturation of testis. No transcripts of *amhy* were detected in any female samples. *amha* was expressed in both sexes starting from 20 dpp to 2.5-year-old adults. Different letters mark the significant differences (*p* < 0.05) of *amha* or *amhy* expression among different developmental stages. Asterisk indicates the significant differences (*p* < 0.05) of expression between *amha* and *amhy* in the same stage. (*e*) Spatial expression of *amha* and *amhy* mRNA in 180 dpp gonads. (*f*) Spatial expression of *amha* and *amhy* mRNA in 1-year-old gonads. (*g*) Spatial expression of *amha* and *amhy* mRNA in 2-year-old gonads. Abbreviations: Sg, spermatogonia; Sc, spermatocytes; St, spermatid; Sz, spermatozoon; Se, Sertoli cells; Oc, oocytes.

A total of 66 published transcriptomes [40] were used for expression analysis of *amh* genes in different tissues of adult *S. schlegelii* *amhy* was predominantly expressed in testes and expressed at a low level in male liver and brain tissue. No transcripts of *amhy* were detected in any female tissues. *amha* displayed significantly higher expression in the gonads compared to other tissues (figure 2*a*). Furthermore, two transcripts of *amha* and two transcripts of *amhy* were also detected from the assembled transcriptomes of ovary and testis tissue. The alignment of transcripts identified a 5 bp ‘CAGAA’ insertion in the seventh, last exon (figure 2*b*). This led to premature transcription termination, which resulted in the lack of TGF-β domain. The expression analysis of the four transcripts showed that the dominant transcript was always the one with complete TGF-β domain (figure 2*c*).

Further, 38 transcriptomes of gonads covering different developmental stages and sex-determining periods were sequenced. *amhy* started to express in male samples at 20 dpp (days post parturition), though at a very low level. Peak expression of *amhy* was detected at 50 dpp during the sex-differentiation period. *amhy* did not show any expression at 90 dpp when male sex was determined (figure 2*d*). No transcripts of *amhy* were detected in any female samples. *amha* was expressed in both sexes starting from 20 dpp to 2.5-year-old adults, with much higher levels observed in mature gonads. In most cases, male samples expressed more *amha* than *amhy* (figure 2*d*).

*In situ* hybridization (ISH) was also performed on histological sections of the gonads of male and female samples at 180 dpp, 1 year old and 2 years old. Given the high similarity of *amhy* and *amha*, common probes of *amhy* and *amha* (marked as *amhy* + *amha*) and *amha*-specific probes were synthesized, respectively. Across different developmental stages of testis tissue, both *amhy* and *amha* were detected in Sertoli cells (figure 2*e–g*). In ovary tissue, *amha* was observed in primary oocytes (figure 2*e*–*g*).

### 2.4. Overexpression of *amhy* caused female-to-male sex reversal in *Sebastes schlegelii*

**Figure 3.**
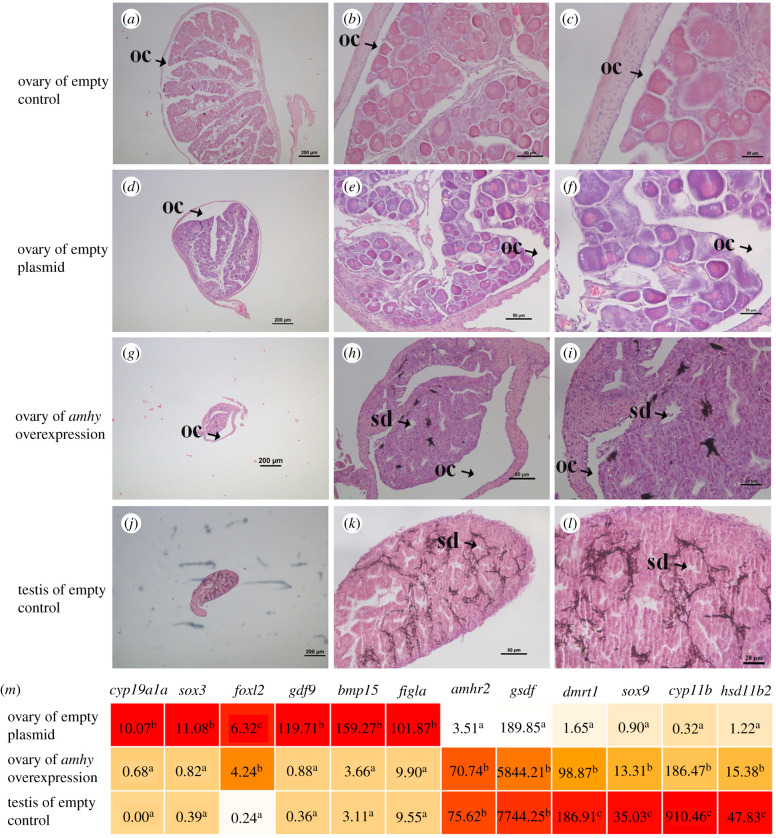
Overexpression of *amhy* caused female-to-male sex reversal. (*a–c*) Histology of an ovary from ­the empty control group; (*d–f*) histology of an ovary from the empty plasmid feeding group; (*g–i*) histology of a testes from the empty control group; (*j–l*) histology of an ovary from the *amhy* overexpression group. Abbreviations: oc: ovary cavity; sd: sperm duct. (*m*) The expression patterns of sex-related genes in gonads of three groups. Numbers represented the TPM of each gene. Different letters indicated significant difference (*p* < 0.05). Abbreviations: *cyp19a1a*: aromatase gonad form; *sox3*: SRY-box transcription factor 3; *foxl2*: ForkheadboxL2; *gdf9*: growth differentiation factor 9; *bmp15*: bone morphogenetic protein 15; *amhr2*: type II anti-müllerian hormone receptor; *gsdf*: gonadal soma-derived factor; *dmrt1*: doublesex and mab-3-related transcription factor 1; *sox9*: SRY-box transcription factor 9; *cyp11b*: 11 beta hydroxylases; *hsd11b2*: 11β-hydroxysteroid dehydrogenase type 2.

*amh* overexpression plasmid feeding can trigger female-to-male transition in orange-spotted grouper (*Epinephelus coioides*) [42,43], which indicated that plasmid feeding is feasible for overexpression experiment in fish. The effect of overexpression of *amhy* in *S. schlegelii* was investigated *in vivo*. 40 dpp fry were divided into three groups. Fry feed with a commercial diet was the empty control. Fry feed with a commercial diet containing the empty plasmid was the empty plasmid control. The *amhy* overexpression group was feed with the commercial diet containing *amhy* overexpression plasmid. The genetically determined sex ratio of the three treatment groups was approximately 1 : 1, with the empty control being 28 female : 32 male, the empty plasmid control 31 female : 29 male, and the *amhy* overexpression group 29 female : 31 male. Histological examination of gonads for 180 dpp revealed that overexpression of *amhy* resulted in incomplete sex reversal for all 29 females, whereas no sex reversal was observed in females belonging to the empty control (*n* = 28) and empty plasmid control groups (*n* = 31)*.* The gonads of genetic females at 180 dpp from the empty control and empty plasmid control groups displayed typical ovary structures, including the ovary cavity, oogonia and primary oocytes (figure 3*a*–*f*). The gonads of genetic males displayed typical testis structure including the sperm duct (figure 3*g–i*). In the group with *amhy* overexpression, the gonads of all genetic females displayed a clear testicular structure with a sperm duct-like cavity (figure 3*j–l*), as well as a clear ovary cavity, which indicated incomplete sex reversal.

Furthermore, the expression profiles of a set of sex-differentiation or sex-specific genes were characterized using eight transcriptomes of gonads from control female, *amhy* overexpression female and normal male at 180 dpp. The expression levels of female-related genes, such as *cyp19a1a*, *sox3*, *foxl2*, *gdf9*, *bmp15* and *figla* were significantly decreased whereas the male-related genes, such as *amhr2*, *gsdf*, *dmrt1*, *sox9*, *cyp11b* and *hsd11b2* were significantly increased in *amhy* overexpression female (figure 3*m*). Moreover, *amhy* overexpression female exhibited similar gene expression patterns to those of normal male, which provided evidence of sex reversal at molecular level.

### 2.5. The origin and phylogenetic analysis of *amh* genes of *Sebastes schlegelii*

**Figure 4.**
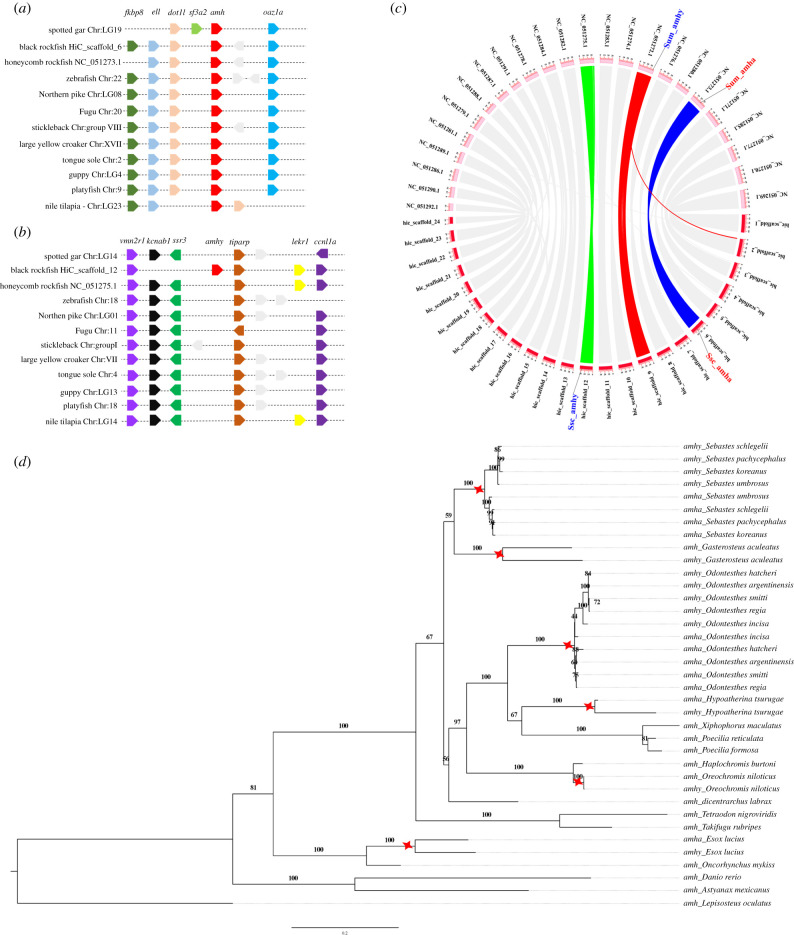
Evolution of *amh* in teleost. (*a*) Synteny analysis of genes adjacent to *amha* among 12 teleost fish genomes. Orthologues of each gene were shown in the same colour. Grey indicated uncharacterized protein-coding genes or lncRNAs. Gene orientation is indicated by the direction of the arrows. (*b*) Synteny analysis using *tiparp* gene as reference in teleost. (*c*) Chromosome collinearity analysis between *S. schlegelii* (Clade C) and *S. umbrosus* (Clade D), where equivalent copies of *amha* and *amhy* are labelled for each species (Ssc and Sum, respectively). (*d*) A maximum-likelihood phylogeny of 37 *amh* protein-coding sequences (both *amha* and *amhy*). The phylogenetic tree was estimated using 1000 bootstraps and bootstrap values are labelled at nodes. The *amh* protein-coding sequence of spotted gar (*Lepisosteus oculatus*) was used as the outgroup, and gene duplication events are illustrated by red stars.

A syntenty map was generated for *amha, amhy* and their adjacent genes to estimate their genomic origins (figure 4*a*,*b*). Eleven teleost species including *S. schlegelii* were used for synteny analysis with spotted gar (*Lepisosteus oculatus*) as the outgroup. The genes adjacent to *amha* were highly conserved in all selected teleosts (figure 4*a*). *amhy* was only present in *S. schlegelii* HiC_scaffold_12, although a group of adjacent genes was conserved among all selected teleost species (figure 4*b*). Two genes (*kcnab1* and *ssr3*) upstream of *S. schlegelii amhy* were absent in other species. These results support HiC_scaffold_6 as the conserved location of the *S. schlegelii* ancestral *amh* gene (*amha*), where *amhy* originated from a duplication of *amha* and followed by translocation to the future sex chromosome, HiC_scaffold_12. Chromosome synteny analysis between *S. schlegelii* and *S. umbrosus* indicated that all the homologous chromosomes showed very high collinearity. It is interesting to see that the chromosomes where *amha* is located (HiC_scaffold_6 in *S. schlegelii* and NC_051273.1 in *S. umbrosus*) showed very high collinearity between these two species (figure 4*c*, highlighted in blue). However, *amhy* is located in two different homologous chromosomes (figure 4*c*, highlighted in green and red).

A maximum-likelihood phylogeny was constructed for 37 protein-coding sequences of *amh* genes (both *amha* and *amhy*). Samples used for phylogenetic reconstruction included reported male-specific duplications of *amh* genes in Patagonian pejerrey [9], northern pike [11], Old World silverside [44] and *Odontesthes* species [20], as well as six *amh* genes identified from three other *Sebastes* species: *S. umbrosus*, *S. koreanus* and *S. pachycephalus* (two genes for each species, respectively; figure 4*d*). In *Sebastes*, all duplicated *amhy* genes clustered together across the four sample species, and this group was then most closely related to the original *amha* genes in the same species (figure 4*d*). The same pattern was also observed for *Odontethes* (figure 4*d*). For the other sample taxa, *amh* genes clustered according to taxonomic identity (i.e. species or genus) with significant bootstrap values (figure 4*d*). Since *amhy* genes did not group across genera, this phylogenetic pattern suggests that the origin of each duplicated sex-specific *amh* gene is independent and lineage-specific.

### 2.6. The duplication of *amh* within the *Sebastes* genus

**Figure 5.**
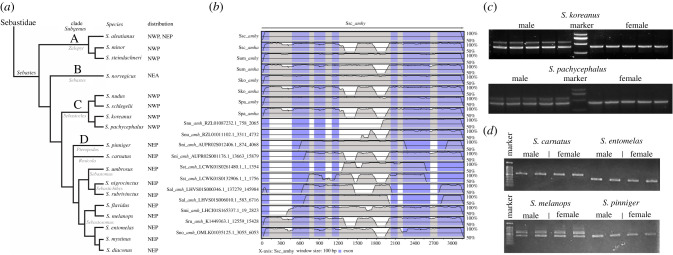
Comparison of *amh* genes among the surveyed *Sebastes* species. (*a*) A cladogram of selected *Sebastes* species used in this study, based on the Bayesian phylogeny produced by Hyde & Vetter [21]. Subgenera contained within each clade are labelled and the geographic region for each species is provided, which includes the northwest Pacific (NWP), northeast Pacific (NEP) and northeast Atlantic (NEA) oceans. (*b*) Two *amh* genes were identified in seven of the 10 *Sebastes* species. The presence or absence of the two insertions in intron 4 distinguishes one as *amhy*-like and the other as *amha*-like. (*c*) PCR amplification in the two northwest Pacific *Sebastes* species related to *S. schlegelii* (Clade C), which produced two bands in all male samples but only one band in female samples. (*d*) PCR amplification in four of the seven tested species of northeast Pacific rockfish (Clade D), which produced either one or two bands for all samples (male or female) of each species.

To further characterize the evolution of *amha* and *amhy* in the genus *Sebastes*, we searched for the orthologous genes in nine published genomes of *Sebastes* species (*S. aleutianus, S. koreanus*, *S. minor*, *S. nigrocinctus*, *S. nudus*, *S. norvegicus*, *S. rubrivinctus*, *S. steindachneri* and *S. umbrosus*). Two species, *S. koreanus* and *S. nudus,* are closely related to *S. schlegelii,* and all three species occur in the northwest Pacific Ocean (Clade C containing the subgenus *Sebastocles*; figure 5*a*) [21]. Three species, including *S. aleutianus* found in the north Pacific Ocean*,* and *S. minor* and *S. steindachneri* from the northwest Pacific Ocean, belong to a separate phylogenetic clade that probably split earlier in the evolution of the *Sebastes* genus (Clade A with the subgenus *Zalopyr*; figure 5*a*) [21]. Three species, *S. nigrocinctus, S. rubrivinctus* and *S. umbrosus*, occur in the Northeast Pacific Ocean and belong to a separate, more derived clade of *Sebastes* that dominates rockfish diversity in that region (Clade D including the subgenera *Pteropodus, Rosicola, Sebastomus, Sebastichthys* and *Sebastosomus*; figure 5*a*) [21]. Finally, *S. norvegicus* occurs in the North Atlantic Ocean and belongs to another clade located between the *S. schlegelii* and *S. aleutianus* clades (Clade B containing the subgenus *Sebastes*; figure 5*a*) [21].

Two *amh* genes were identified from six of the nine *Sebastes* species (figure 5*b*). Only one *amh* gene was detected from the *S. norvegicus* (Clade B), *S. minor* (Clade A) and *S. rubrivinctus* (Clade D) genome assemblies. It should be noted that none of these species are closely related to *S. schlegelii* (Clade C), which potentially suggests divergence within *Sebastes* for the *amhy* gene. The putative *amhy* gene was detected in the six remaining species (spread across Clades A, C and D) and all species contained the 166 bp insertion located on intron 4. Notably, both *amh* genes contained the two insertions in *S. aleutianus* (Clade A), whereas both *amh* genes only contained the 131 bp insertion in *S. umbrosus* (Clade D). However, when comparing the coding sequence, the two genes of *S. aleutianus* corresponded to *amha-like* and *amhy-like*, respectively (electronic supplementary material, figure S3). It is difficult to determine whether this difference in *S. aleuntianus* is caused by an incorrect assembly of the genome region, or if the *amha* gene in *S. aleutianus* does indeed include these two insertions.

The alignment of all the identified *amh* genes (sometimes with incomplete sequences) indicated that the primers designed for SD in *S. schlegelii* could be successfully applied to other *Sebastes* species. We tested the feasibility of these primers as a sex identification assay using two species of northwest Pacific rockfish that are both related to *S. schlegelii*—*S. koreanus* and *S. pachycephalus* (Clade C)—as well as seven species of distantly related northeast Pacific rockfish: *S. carnatus*, *S. diaconus, S. entomelas*, *S. flavidus, S. melanops*, *S. mystinus* and *S. pinniger* (Clade D). In the northwest Pacific rockfish species, PCR amplification results matched for *S. schlegelii*, with two bands in males and one band in females (figure 5*c*). Sequencing of the *amhy* and *amha* PCR products in these two species confirmed the occurrence of one insertion of 166 bp in intron 4, as well as highly conserved intron nucleotide sequences (electronic supplementary material, figure S4). By contrast, in the northeast Pacific rockfish, PCR amplification produced one or two bands for all samples of each species, and males and females were not distinguished (figure 5*d*). These results indicate that the *amhy* gene is not sex-dependent among northeast Pacific rockfish in Clade D.

## 3. Discussion

Male-specific duplication of *amh* has been proved to be conserved in two clades of teleost fish, namely northern pike [11] and among *Odontesthes* silversides [20]. This work provides a third example of an *amh* duplication event, within a clade of *Sebastes* rockfish. A phylogenetic analysis suggests that male-specific *amhy* genes have evolved independently within each teleost lineage. The repeated, independent recruitment of the same gene for SD supports the ‘limited options’ hypothesis for the evolution of genetic SD mechanisms [45].

We observe that the scale of genetic divergence between the *amha* and *amhy* paralogs varies across species. The northern pike shows the highest degree of sequence divergence between two paralogs, with an average of 79.6% genomic sequence identity [11]. In Nile tilapia, *amhy* and *amha* only differs by one SNP [10]. The shared identity between the two paralogues of Patagonian pejerrey ranges from 89.1% to 100% depending on the exon [9]. In *S. schlegelii*, *amha* and *amhy* share high-nucleotide identity ranging from 91.8% to 97.3% between exon sequences. The major differences are two insertions in *amhy* intron 4. The sequence divergence between the *amhy* and *amha* paralogs in species may be an indicator of duplication history or the selection pressure upon the sex-determining genes during evolution. It is noteworthy that compared to *amha*, the duplicated *amhy* always contains insertions in the introns, such as 557 bp insertion in intron 3 in Patagonian pejerrey [9], 396 bp insertion in intron 1 in northern pike [11], 195 bp insertion in intron 1 in the Old World silverside [44], and approximately 0.5 kb insertion in intron 3 in the genus *Odontesthes* [20]. Introns 1 and 3 appear to be hotspots for insertions. It would be interesting to see if the intron insertions play some functional roles.

In some special events, the duplicated *amhy* loses some exons or conserved domains. For example, in the case of *Hypoatherina tsurugae*, *amhy* lacks exons 2 and 3 but contains a complete TGF-β domain, suggesting the ability of binding to its receptor *amhr2* and then activating the downstream signalling of testis differentiation [44]. In Nile tilapia, a tandem duplication caused two copies of *amhy* in the Y chromosome, one of which contained 5 bp (ATGTC) insertion in the exon 6, producing a protein lacking TGF-β domain, which was regarded as the degenerative gene named *amhΔy* [10]. A recent study reported that the association of *amhΔy* with sex was more conserved than the missense SNP of *amh* in different Nile tilapia strains [19]. We also observed ‘truncated’ transcripts produced by alternative splicing from *S. schlegelii*. Two alterative transcripts were detected both for *amha* and *amhy* in *S. schlegelii*. It is probable that *amhy* still keeps the same alternative splicing mechanism with *amha* after duplication and translocation. Interestingly, Nile tilapia produced three copies of *amh* genes to create one ‘truncated’ protein (Amh*Δ*y), whereas *S. schlegelii* took the alternative splicing strategy to produce the ‘truncated’ protein. This Amh*Δ*y protein in Nile tilapia lacking the TGF-β domain cannot directly bind to *amhr2* [10]. Further investigation of how such ‘truncated’ proteins participate in testicular development and how the two *amh* genes cooperate to initiate testicular differentiation of *S. schlegelii* needs to be explored further.

In several species like Patagonian pejerrey [9], Nile tilapia [10] and northern pike, the duplicated *amh* gene has been reported to be male-specific and has been validated to be the MSD gene. The *amhy* identified in *S. schlegelii* is also male-specific and can drive the testis differentiation cascade. A previous study reported that morphological differentiation of *S. schlegelii* ovaries and testes was not synchronous, with ovary differentiation occurring at approximately 25 dpp and testis differentiation at approximately 85 dpp [29]. In our study, *amhy* started to be expressed at 20 dpp prior to the morphological differentiation of ovaries and testes in *S. schlegelii*. This pattern matches results for Nile tilapia [10] and northern pike [11], suggesting that the putative function of *amhy* is to suppress ovary development in genetic males. RNA-seq analysis and ISH in different development stage testis indicated that both *amhy* and *amha* were expressed in Sertoli cells. These results agreed with previously reported results in medaka [46], zebrafish [47], Japanese eel [48] and Japanese flounder [49], which indicates the conserved role of *amh* in testis differentiation among teleosts. Additionally, the *amh* gene expression has been recorded in follicular cells and its expression seems to be specific to granulosa cells in medaka [46] and zebrafish [47]. This appears to be conserved even among mammals [50,51]. However, *amha* was detected in primary oocytes in ovaries at different developmental stages in *S. schlegelii*, indicating that *amha* may play some roles in oocytes maturation, which differs from reports for other teleosts and mammals. However, the exact roles of *amh* in this regard need in-depth observation.

Previous studies have conducted overexpression assays to investigate the function of putative MSD genes in SD. In the medaka *Oryzias latipes*, overexpression of *DMY* cDNA controlled by the CMV promoter using pIRES-hrGFP-1a vector, caused XX sex reversal [52]*.* In another medaka species, *O. luzonensis,* the presence of a genomic fragment that included *Gsdf^Y^* also caused XX sex reversal [53]*.* Overexpression of the duplicated *amh* gene in Nile tilapia [10] and northern pike [11] resulted in sex reversal in both species. In this study, *amhy* overexpression resulted in female-to-male sex reversal for all tested genetic females, which indicated that the *amhy* protein was sufficient to trigger testicular development in *S. schlegelii*. Gene expression analysis of sex-reversed female suggested that *amhy* determined the sex of *S. schlegelii* probably by suppressing gonadal aromatase expression and/or activating a male-specific signalling pathway. These results provided sufficient evidence to support *amhy* as the MSD gene in *S. schlegelii*.

The *amhy* PCR assay developed here can be successfully applied in at least two other northwest Pacific rockfish species closely related to *S. schlegelii* (Clade C), but it was not successful for distinguishing males and females in at least one major clade of *Sebastes* found in the northeast Pacific Ocean (Clade D). This pattern in our results indicates that the *amhy* MSD gene may not be universal among *Sebastes*. The *amhy* gene may be the ancestral MSD gene in *Sebastes*, which has been lost by the clade of northeast Pacific rockfish (Clade D). Alternatively, *amhy* may have evolved as the MSD gene in only the clade of northwest Pacific rockfish that contains *S. schlegelii* (Clade C). If it is the latter case, *amh* duplication happened in the ancestral genome of *Sebastes*, but the gene was translocated to different positions in the genome for different clades of rockfish, based on the observation that *amhy* was found on two different homologous chromosomes in *S. schlegelii* and *S. umbrosus*. Obviously, *amhy* is the sex-determining gene in *S. schlegelii* but not in *S. umbrosus*. We suspect that the translocated position determined whether the translocated *amhy* became the sex-determining gene or not. Further PCR assays and sequencing results are required from a wider diversity of species to determine the representation of the *amhy* MSD among *Sebastes* rockfish. The developed PCR assay has the potential to improve fisheries management and conservation in *S. schlegelii* and closely related species including *S. koreanus* and *S. pachycephalus*. Using this assay, the sex of individuals can be genetically identified at any developmental stage without relying on the examination of urogenital papillae in sexually mature adults, or the lethal dissection of gonads. This discovery will aid stock assessment efforts in aquaculture, and any future population genetic research.

In conclusion, we identified a duplication of *amh* in *S. schlegelii,* which generated a male-specific copy named *amhy*. We revealed that *amhy* was essential for male SD in *S. schlegelii* and provided substantial evidence to support *amhy* as the MSD gene. We hypothesized that the GSD using *amhy* was conserved in the clade of northwest Pacific rockfish (Clade C), and we developed an effective and efficient sex marker for this group. An *amh* MSD gene may therefore be the ancestral state of *Sebastes*, which has been subsequently lost in the clade of northeast Pacific rockfish, or it may have evolved specifically among northwest Pacific rockfish.

## 4. Material and methods

### 4.1. Samples

Fifty specimens of *S. schlegelii* (body length: 20.3 ± 1.5 cm, weight: 261.5 g ± 25.0 g) were captured from a deep-sea cage in Zhucha Island (Qingdao, Shandong, China) and then transported to the laboratory at Ocean University of China. Fish were cultured in the laboratory for 3 days before dissection. Gonads were dissected to determine the physiological sex of each individual. A piece of muscle tissue was fixed in 95% ethanol. Ten male samples and ten female samples were selected for resequencing. The fry of *S. schlegelii* were obtained from 3-year-old brood stock and cultured in Weihai Taifeng Hatchery Co., Rushan, China. Thirty fry were sampled every 10 days starting at 20 dpp until 90 dpp. Considering that the gonads were too small to be isolated, the entire trunks were fixed in RNA-later for RNA isolation. Meanwhile, muscle tissue from each sample was fixed in 95% ethanol for DNA extraction and further genetic sex identification. Gonads from different developmental stages were sampled from 180 dpp, 200 dpp, 1-year-old, 1.5-year-old and 2-year-old individuals cultured in Weihai Yinze Biotechnolgy Co., Wendeng, China. Twelve individuals (six male and six female) were sampled for each stage. One piece of gonad was immediately frozen in liquid nitrogen and stored at −80°C for RNA extraction. The other piece of gonad was fixed in 4% paraformaldehyde (PFA) for 24 h at 4°C and then dehydrated with methanol. Samples from three northwest Pacific rockfish species (*S. schlegelii*, *S. koreanus* and *S. pachycephalus*; all belonging to Clade C; figure 5*a*) and seven species of northeast Pacific rockfish species (*S. carnatus, S. diaconus, S. entomelas, S. flavidus, S. melanops, S. mystinus* and *S. pinniger*; all in Clade D; figure 5*a*) were used to test the efficiency and effectiveness of the sex marker. The northwest Pacific species samples were bought from the Xuejiadao Seafood Market in Qingdao, China. A total of 40 individuals (20 males and 20 females) for *S. schlegelii,* 23 individuals for *S. koreanus* (10 males and 13 females) and 32 individuals for *S. pachycephalus* (15 males and 17 females) were used for validation. Samples of *S. carnatus* were collected as part of a previous study from waters off southern California [31], and the remaining six northeast Pacific samples were collected off Oregon by the Oregon Department of Fish and Wildlife. Full sampling information is provided in the electronic supplementary material for a previous study that used the same samples [32]. Two males and two females were used for each northeast Pacific species, except for *S. mystinus* where only one male and one female were used (electronic supplementary material, figure S5).

### 4.2. Resequencing and coverage analysis

Genomic DNA was extracted from muscle of *S. schlegelii* using Tris-Phenol method and subjected to quality control. An input amount of 1μg high-quality DNA was used for the WGS library construction using MGIEasy DNA Rapid Library Prep Kit (BGI, catalog no. 1000006985), and 100 bp paired-end reads were generated on an DIPSEQ T1 platform. Raw reads were cleaned using SOAPnuke [54] to remove adapter sequences and low-quality reads. Clean reads were mapped to the reference genome of *S. schlegelii* using BWA [55] with default parameters. Samtools v. 1.4 [56] was then used to calculate coverage depth of scaffolds for each sample. Coverage depth was normalized with log_2_(coverage depth value) and then used to compare the difference between sexes with a sliding window of 1000 bp. We added 1 to each value to avoid infinitely high numbers associated with log_2_ 0.

### 4.3. Sequence analysis of *amha* and *amhy*

Shared identity between *amha* and *amhy* gene exon sequences and protein sequences of *S. schlegelii* was calculated using EMBOSS Water [57] implemented on EMBL-EBI [58,59] with default parameters. The signal peptide and conserved domains of *amh* genes were annotated using SMART [60]. BLAST (blastn version 2.2.26) was used to identify *amh* genes from the genome of nine *Sebastes* genus species (assembly ID: SRub1.0 for *S. rubrivinctus*, fSebUmb1.pri for *S. umbrosus*, ASM191080v2 for *S. aleutianus*, ASM191078v2 for *S. steindachneri*, ASM191076v2 for *S. minor*, ASM433533v1 for *S. koreanus*, ASM47523v3 for *S. nigrocinctus*, ASM433536v1 for *S. nudus* and ASM90030265v1 for *S. norvegicus*) with e-value of 2 × 10^−5^ and alignment length no less than 500 bp. The alignments of the *amh* genes of *S. schlegelii* and incomplete *amh* genes of other *Sebastes* genus species were performed and visualized using the mVISTA Shuffle-LAGAN program [61,62] with default parameters.

### 4.4. Expression analysis of *amha* and *amhy*

A total of 104 transcriptomes, 66 of which are available at CNSA (CNGB Nucleotide Sequence Archive) under the accession ID CNP0000222 [40] and 38 newly built libraries covering sex-determining period (20, 30, 50, 70 and 90 dpp) and different developmental stages of gonads (200 dpp, 1.5 years old) were used to analyse the expression of two *amh* genes. These new libraries were sequenced 150 bp from each end using the NovaSeq 6000 platform. Basic statistics of the 38 transcriptomes were listed in electronic supplementary material, table S3. TPM (transcripts per kilobase million), a more accurate measure of RNA abundance than RPKM (reads per kilobase million) [63], was calculated using Salmon version 0.7.2 [64] with default parameters and visualized using GraphPad Prism 7. To compare the expression of *amha* or *amhy* among different tissues or different developmental stages, pair-wise comparison of gene counts was performed by DEseq 2 [65]. *p*-value of each comparison was extracted for *amha* and *amhy*. Differences were considered significant when *p* < 0.05. To detect the expression differences between *amha* and *amhy* in the same tissue or the same stage, independent *t*-test was conducted by SPSS (V. 20.0.0). Differences were considered significant when *p* < 0.05.

### 4.5. Sex marker developed to distinguish genetical male and female in genus *Sebastes*

A pair of primers (Fw5′-GTAAACCAAGAACTGAGGAGGAG-3′, Rv5′-GAGAAAAGCAGAAGTGGAATCA-3′, also shown in electronic supplementary material, table S4) spanning the 166 bp insertion in *amhy* intron 4 was designed for PCR amplification in male and female *S. schlegelii* samples. The following PCR amplification program was carried out: 5 min at 95°C, 32 cycles of 30 s at 95°C, 30 s at 57°C, 40 s at 72°C per cycle, 5 min at 72°C, held at 4°C. PCR amplification for the validation of the applicability of the primers in *S. koreanus*, *S. pachycephalus* and the northeast Pacific species (*S. carnatus, S. diaconus, S. entomelas, S. flavidus, S. melanops, S. mystinus* and *S. pinniger*) was also performed with the same program. PCR products were detected by 1.5% or 1.6% agarose gel electrophoresis.

### 4.6. Phylogenetic and synteny analyses

A set of *amh* protein-coding sequences were collected and retrieved from NCBI or the Ensembl database (accession numbers listed in the electronic supplementary material, table S1). The alignment of the *amh* proteins sequences was performed using MAFFT v. 7.475 [66] and a maximum-likelihood phylogeny was constructed using IQ-TREE version 1.6.12 with 1000 bootstraps [67]. Genomicus version 102.01, a synteny browser (https://www.genomicus.biologie.ens.fr/ [68,69]), was used to generate the synteny sketch map of spotted gar, zebrafish (*Danio rerio*), northern pike, fugu (*Takifugu rubripes*), threespine stickleback (*Gasterosteus aculeatus*), Nile tilapia, platyfish (*Xiphophorus maculatus*), large yellow croaker (*Larimichthys crocea*), tongue sole (*Cynoglossus semilaevis*) and guppy (*Poecilia reticulata*). The synteny sketch map of *S. schlegelii* was generated from the published genome [40] (accession ID CNP0000222) according to the annotated genes based on their location. The synteny sketch map of *S. umbrosus* was also generated from the published genome (assembly id: fSebUmb1.pri). Chromosome-scale synteny analysis of *S. schlegelii* and *S. umbrosus* was performed using MCScanX [70] and visualized using TBtools v. 1.075 [71]. Protein-coding genes were used for synteny analysis and only the best BLAST results were retained.

### 4.7. *In situ* hybridization

ISH of testis and ovary was performed as previously described [72]. The probes of *amha* and that of *amhy* and *amha* were amplified, respectively, from cDNA using two pairs of primers list in the electronic supplementary material, table S4. The results were imaged by AZ100 (Nikon, Tokyo, Japan).

### 4.8. Overexpression of *amhy* in fry of *Sebastes schlegelii*

The overexpression vector was constructed as described in a previous study [10]. Shortly, the *amhy* ORF was subcloned into the multiple cloning sites downstream of the CMV promoter of pIRES-hrGFP-1a vector. Then, the plasmids were extracted and diluted to 10 µg/µl. The procedures of plasmids packaging and feeding were similar to the studies in orange-spotted grouper (*Epinephelus coioides*) [42,43] with some differences. Briefly, the empty plasmids (pIRES-hrGFP-1a) and *amhy* overexpression plasmids (pIRES-hrGFP-1a-amhy) were encapsulated by liposome6000 (Beyotime) at the volume ratio of 1 : 1, making the final concentration of construct 5 µg µl^−1^. The constructs were then mixed with a commercial diet at the ratio of 1 ml kg^−1^ diet; 1500 40 dpp fry were randomly selected and divided into three groups: empty control (*n* = 500), control group (*n* = 500) and *amhy* overexpression group (*n* = 500). These three groups were cultured in separate tanks with different feeds but same amount. The empty control was fed with a normal commercial diet. The control group was fed with a diet containing empty plasmids. The *amhy* overexpression group was fed with a diet containing *amhy* overexpression plasmids. The treatment lasted 50 days. Sixty individuals were randomly selected from each group and sacrificed at 180 dpp (90 days after the completion of treatment). Gonads and muscles were sampled for histological analysis. Physiological sex for each individual was determined by the morphology of gonads and routine hematoxylin-eosin staining. The genetic sex of these samples was determined using the sex marker developed in this study. Gonads from control female, *amhy* overexpression female and normal male were also sampled in triplicates for the following RNA extraction and transcriptome libraries construction. Gene expression and statistical analysis were carried out as described above.

## Data accessibility

The resequencing data and transcriptome data were submitted to NCBI Sequence Read Archive (SRA) under the project number PRJNA656655. *amha* and *amhy* sequences of *S. schlegelii*, *S. koreanus* and *S. pachycephalus* have been submitted to GenBank with accession numbers MW591738–MW591743.
